# The Effect of Axial Length on the Thickness of Intraretinal Layers of the Macula

**DOI:** 10.1371/journal.pone.0142383

**Published:** 2015-11-06

**Authors:** Andrea Szigeti, Erika Tátrai, Boglárka Enikő Varga, Anna Szamosi, Delia Cabrera DeBuc, Zoltán Zsolt Nagy, János Németh, Gábor Márk Somfai

**Affiliations:** 1 Department of Ophthalmology, Semmelweis University, Budapest, Hungary; 2 Bascom Palmer Eye Institute, University of Miami Miller School of Medicine, Miami, Florida, United States of America; Massachusetts Eye & Ear Infirmary, Harvard Medical School, UNITED STATES

## Abstract

**Purpose:**

The aim of this study was to evaluate the effect of axial length (AL) on the thickness of intraretinal layers in the macula using optical coherence tomography (OCT) image analysis.

**Methods:**

Fifty three randomly selected eyes of 53 healthy subjects were recruited for this study. The median age of the participants was 29 years (range: 6 to 67 years). AL was measured for each eye using a Lenstar LS 900 device. OCT imaging of the macula was also performed by Stratus OCT. OCTRIMA software was used to process the raw OCT scans and to determine the weighted mean thickness of 6 intraretinal layers and the total retina. Partial correlation test was performed to assess the correlation between the AL and the thickness values.

**Results:**

Total retinal thickness showed moderate negative correlation with AL (r = -0.378, p = 0.0007), while no correlation was observed between the thickness of the retinal nerve fiber layer (RNFL), ganglion cell layer (GCC), retinal pigment epithelium (RPE) and AL. Moderate negative correlation was observed also between the thickness of the ganglion cell layer and inner plexiform layer complex (GCL+IPL), inner nuclear layer (INL), outer plexiform layer (OPL), outer nuclear layer (ONL) and AL which were more pronounced in the peripheral ring (r = -0.402, p = 0.004; r = -0.429, p = 0.002; r = -0.360, p = 0.01; r = -0.448, p = 0.001).

**Conclusions:**

Our results have shown that the thickness of the nuclear layers and the total retina is correlated with AL. The reason underlying this could be the lateral stretching capability of these layers; however, further research is warranted to prove this theory. Our results suggest that the effect of AL on retinal layers should be taken into account in future studies.

## Introduction

Since its first ophthalmic application in the 1950s, ultrasound (US) has become a standard diagnostic method in ophthalmology. The main advantage of this non-invasive modality, when compared with existing ophthalmological examination techniques was that it allowed not only qualitative but also quantitative evaluation of the eyeball; i.e. it enabled the measurement of the axial length (AL) of the eye and the thickness of the ocular wall. As a result of continuous improvement, high-resolution imaging and investigation of the ocular and orbital blood flow characteristics can also be performed using US [1, 2].

The first paper describing the measurement of the ocular coat dimensions using US and the correlation between the thickness of the ocular wall and AL was published in 1984 [3]. Eight years later, Németh et al showed that the volume of the ocular coats is nearly constant in healthy eyes; furthermore, their results confirmed that the thickness of the ocular wall correlates negatively with the AL of the eye [4]. On the other hand, in eyes with uveitis, hypotonia or exophthalmus, the thickness and volume of the ocular wall were increased, as a result of the edema, while in eyes with glaucoma both the thickness and volume of the ocular wall were decreased, probably as a consequence of the destruction of the ganglion cells [4].

In the early 1990s, the development of optical coherence tomography (OCT) enabled high-resolution cross-sectional imaging of the retina in vivo along with the quantitative analysis of retinal thickness, enabling accurate diagnosis and follow-up of various retinal pathologies. OCT rapidly became one of the most frequently used imaging tools in ophthalmological practice and nowadays it plays an essential role in the diagnosis and follow-up of several retinal diseases and also in the guiding of therapeutic decisions [5].

Due to the high resolution of OCT images and the different optical properties of the intraretinal structures, various layers of the retina can be detected on OCT images. Several softwares have been developed in the last few years in order to allow the separate measurement of the thickness of the retinal layers, facilitating the localization of structural changes of the retina and the detection of even small changes [6–9]. One of the first image processing softwares, Optical Coherence Tomography Retinal IMage Analysis (OCTRIMA) was developed by Cabrera et al. for time-domain OCT (TD-OCT) images [6]. By the processing of macular OCT images, OCTRIMA enables the detection of the boundaries of several intraretinal layers and the quantitative analysis of the retinal structure [6].

Previous studies suggested that there is a correlation between retinal thickness and AL, age, OCT image quality, gender or race in healthy eyes [10–16]. This study evaluated the effect of AL on the thickness of the total retina and the thickness of the intraretinal layers in healthy eyes using OCT image segmentation.

## Patients and Methods

This cross-sectional study was performed at the Department of Ophthalmology, Semmelweis University, Budapest, Hungary. Eighty randomly selected eyes of 70 randomly selected healthy Caucasian subjects (35 male/ 35 female) were enrolled consecutively as they presented at the Optometry outpatient unit of the Department of Ophthalmology, Semmelweis University, between January 2010 and March 2010. All patients were referring either for spectacle prescription, a general ophthalmic follow-up or for a sports activity certification or driving license certification. The eligibility criteria for the participants were best-corrected Snellen visual acuity (BCVA) of 20/20 and the lack of any ocular or systemic diseases, except for controlled hypertension. After the exclusion of subjects with cooperation problems during axial length measurements (mostly children) and eyes where scans were not obtained with proper signal strength enabling the visualization of all layers (Signal Strength <6) we had 53 eyes enrolled in the study.

All participants were treated in accordance with the tenets of the Declaration of Helsinki. Institutional Review Board approval was obtained for all study protocols (Semmelweis University Regional and Institutional Committee of Sciences and Research Ethics). Written informed consent was obtained from all participants in this study. In case of subjects under 18 years of age, written informed consent was obtained from a parent or a legal guardian.

All volunteers underwent an ophthalmic examination including BCVA (measured with Snellen chart adjusted at 5 m, converted to logMAR values for analysis), manifest spherical equivalent refraction (MRSE was defined as the spherical power plus half of the minus cylindrical power (sphere+ ½ cylinder), assessment of intraocular pressure, slit lamp biomicroscopy and binocular ophthalmoscopy with pupil dilation. AL was measured in each eye using a LenStar LS 900 device (LS 900^®^ Haag-Streit AG, Koeniz, Switzerland), which based on the principles of optical low-coherence reflectometry. The instrument uses a broadband superluminescent diode light source (peak wavelength 820 nm) to provide a series of axial biometric dimensions along the line of sight with a clinical accuracy of 12 μm [17, 18]. The measurement wavelength and bandwidth of the instrument equate to an axial resolution of ~ 10 μm, using the formulas from Tanna et al. [19]. A minimum of 5 measurements were obtained for every parameter in each eye for calculating mean values. The same person performed all the measurements.

**Fig 1:**
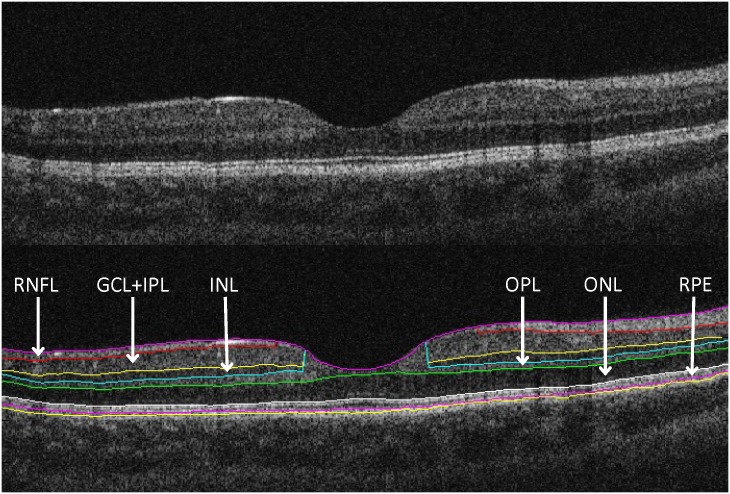
Macular OCT image segmentation using OCTRIMA (A) The image of a healthy macula scanned by the Stratus OCT “Macular Thickness Map” protocol. (B) The same OCT scan processed with OCTRIMA. For the abbreviations see Table 2.

OCT examination was performed using the “Macular Thickness Map” protocol of a Stratus OCT device (Carl Zeiss Meditec, Dublin, CA, USA). The raw macular OCT data were exported from the OCT device and further processed using a computer-aided grading methodology for OCT retinal image analysis (OCTRIMA) described in detail previously by Cabrera et al. [6]. In short, the software is capable to detect intraretinal layers automatically, with the possibility for manual correction of any pitfalls in layer detection [6]. (Fig 1) In order to provide the best possible segmentation results, only those eyes were enrolled in the study where scans were obtained with proper signal strength enabling the visualization of all layers [20].

**Fig 2:**
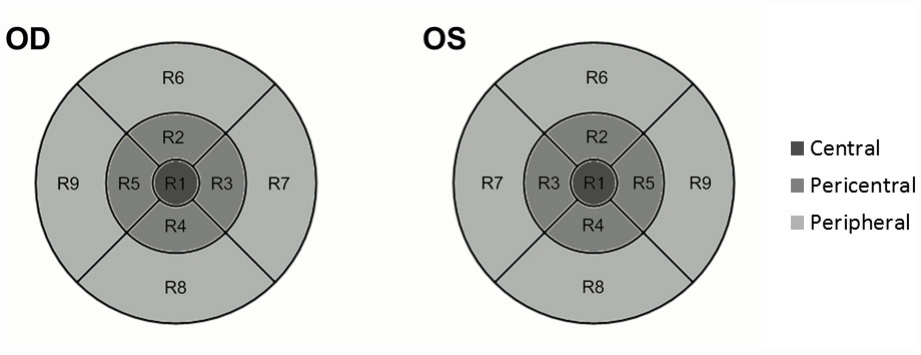
Nine macular regions, defined by the Early Treatment Diabetic Retinopathy Study were analyzed in both eyes by OCT examinations.

The thickness of the total retina and the intraretinal layers were measured in nine macular regions, defined by the Early Treatment Diabetic Retinopathy Study (ETDRS) [21] (Fig 2). Since the number of sampling points is different at the central (R1), pericentral (R2-R5) peripheral (R6-R9) regions because of the radial spoke pattern used in the scanning protocol of Stratus OCT, instead of averaging retinal thickness results in the 9 macular regions, a weighted mean thickness (WMT) was calculated using the following equation, as advised by Massin et al. [22]:

$$WMT=\frac{R1}{36}+\frac{R2+R3+R4+R5}{18}+\frac{(R6+R7+R8+R9)x3}{16}$$

The WMT values for the retinal nerve fiber layer (RNFL), ganglion cell layer and inner plexiform layer complex (GCL+IPL), inner nuclear layer (INL), outer plexiform layer (OPL), outer nuclear layer (ONL), retinal pigment epithelium layer (RPE) and the total retina were obtained for each eye. We also calculated the mean thickness values in the central region and the pericentral and peripheral rings for statistical analyses. Because of anatomical considerations only the ONL and RPE thickness data were used in the central region.

The circumpapillary RNFL thickness was measured by the software of the Stratus OCT device based on the slow “RNFL Thickness Map” protocol.

Statistical analyses were performed using SPSS 16.0 software (SPSS Inc., Chicago, IL, USA) software while power analyses were carried out by the SAS Software version 9.22 (SAS Institute, Cary, NC). The partial correlation test was used to determine the effect of AL on individual layer thickness with age [10], image quality [11, 15, 16], and sex [23–27] as confounders, since these parameters are known to influence OCT thickness measurements.

The STROBE guidelines were followed for the interpretation of the study design and study data. [28] A p value of <0.05 was considered statistically significant.

## Results

Demographic and ocular features of the study population are presented in Table 1.

**Table 1:** Demographic and ocular features of the study subjects.

	Number	Mean ± SD [range]
**Age (years)**		
<20	18	12 ± 4
20–29	12	26 ± 2
30–39	11	32 ± 3
40–50	2	45 ± 0
>50	10	59 ± 5
Total	53	29 ± 17 [6–67]
**MRSE (D)**		
< -4.00	0	NA
-4.00 - -2.25	2	-3.07 ± 0.45
-2.00 - -0.25	12	-0.86 ± 0.63
0.00 - +1.75	25	0.80 ± 0.63
+2.00 - +3.75	8	2.73 ± 0.48
> +4.00	6	5.31 ± 1.01
Total	53	1.08 ± 2.11 [-3.38 - +6.88]
**AL (mm)**		
20.00–21.99	7	21.62 ± 0.36
22.00–23.99	34	22.88 ± 0.58
24.00–25.99	12	24.48 ± 0.36
Total	53	23.07 ± 1.01 [20.95–25.17]
**Eye**		
OD	35	NA
OS	18	NA
**Sex**		
Male	21	NA
Female	32	NA

Abbreviations: MRSE = Manifest refraction in spherical equivalent; AL = axial length.

Table 2 shows the mean layer thickness measurements of the individual retinal layers of the subjects in the central fovea (only outer retinal layer thicknesses), pericentral and peripheral rings (all retinal layers) and the correlation between these layers and AL, adjusted for age, sex and signal strength value.

**Table 2:** Correlations of AL with thickness of macular layers, with both unadjusted data and data after adjusting for age, signal strength value and sex. (n = 53) Whole: entire ETDRS area, fovea: central 1mm ring, pericentral ring: 1–3 mm diameter around the fovea, peripheral ring: 3-6mm diameter around the fovea. The statistical analysis was performed using partial correlation coefficient. Note that due to the foveal anatomy only the ONL, RPE and total retinal thickness results in the foveal region are displayed here.

Macular layer	Mean ± SD (μm)	Unadjusted correlation		Partial correaltion	
		**R**	**P**	**R**	**P**
**RNFL**					
Whole	36.38 ± 2.48	0.167	0.232	0.169	0.241
pericentral ring	23.88 ± 2.47	0.238	0.086	0.222	0.121
peripheral ring	41.49 ± 2.99	0.137	0.329	0.138	0.338
**GCL+IPL**					
Whole	70.42 ± 5.62	-0.310	**0.024**	-0.328	**0.020**
pericentral ring	94.79 ± 6.47	0.036	0.796	0.015	0.919
peripheral ring	65.85 ± 6.12	-0.387	**0.004**	-0.402	**0.004**
**GCC**					
Whole	106.80 ± 7.08	-0.188	0.178	-0.199	0.166
pericentral ring	118.66 ± 7.65	0.108	0.443	0.086	0.552
peripheral ring	107.34 ± 7.76	-0.253	0.068	-0.262	0.066
**INL**					
Whole	33.92 ± 1.94	-0.319	**0.020**	-0.321	**0.023**
pericentral ring	38.49 ± 2.52	0.087	0.534	0.121	0.402
peripheral ring	33.81 ± 2.14	-0.418	**0.002**	-0.429	**0.002**
**OPL**					
Whole	32.36 ± 1.53	-0.290	**0.035**	-0.277	0.051
pericentral ring	37.99 ± 2.37	0.009	0.948	0.004	0.980
peripheral ring	31.90 ± 1.57	-0.369	**0.007**	-0.360	**0.010**
**ONL**					
Whole	81.44 ± 5.68	-0.318	**0.005**	-0.399	**0.004**
Fovea	118.43 ± 9.69	-0.150	0.282	-0.119	0.409
pericentral ring	90.53 ± 7.95	-0.310	**0.022**	-0.330	**0.019**
peripheral ring	77.51 ± 5.33	-0.426	**0.001**	-0.448	**0.001**
**RPE**					
Whole	12.20 ± 1.49	0.130	0.925	0.063	0.665
Fovea	14.56 ± 1.65	-0.234	0.092	-0.212	0.140
pericentral ring	11.90 ± 1.93	-0.242	0.081	-0.214	0.135
peripheral ring	12.17 ± 1.48	0.058	0.680	0.140	0.333
**Total retina**					
Whole	292.23 ± 12.49	-0.383	**0.005**	-0.378	**0.007**
Fovea	237.13 ± 19.55	0.108	0.442	0.148	0.304
pericentral ring	321.82 ± 13.39	-0.112	0.424	-0.114	0.431
peripheral ring	285.55 ± 13.09	-0.456	**0.001**	-0.450	**0.001**
**cpRNFL**	102.88 ± 7.73	-0.198	0.204	-0.171	0.290

Abbreviations: AL: axial length, RNFL: retinal nerve fiber layer, GCL+IPL: ganglion cell and inner plexiform layer complex, INL: inner nuclear layer, OPL: outer plexiform layer, ONL: outer plexiform layer, RPE: retinal pigment epithelium, cpRNFL: circumpapillary retinal nerve fiber layer.

Total retinal thickness showed a moderate negative correlation with AL (p = 0.007, r = -0.378). There was no correlation between AL and the thickness of the RNFL, GCC and RPE (p>0.05, see Table 2). Moderate negative correlation was observed between AL and the “whole” thickness of the GCL+IPL, INL, ONL and total retinal thickness with a more pronounced negative correlation in the peripheral rings of these layers. Also, the peripheral ring of the OPL correlated inversely with AL. There was no correlation between cpRNFL and AL.

## Discussion

This study evaluated the correlation between axial length and the thickness of intraretinal layers in the macula. Our results showed that in the macular area the thickness of the retina and all intraretinal layers, except for the RNFL, GCC and the RPE, correlated with AL with an increasing trend towards the outer layers in the peripheral ring which suggests that the outer layers are elongating or “stretching” with increasing eyeball length. Since the OCT examination was carried out in the macula in a limited, 6 mm diameter wide retinal area, we did not have the opportunity to measure the total volume of the retinal layers involving the entire retina to the ora serrata and thus we were not able to get comparable results to those published by Németh et al. using ultrasound [4].

Conflicting results were reported in previous studies about the correlation between AL and thickness of the intraretinal layers. Cheung et al. measured the thickness of the peripapillary nerve fiber layer and other characteristic parameters of the optic disc (like the area of the optic nerve head, the area of the rim area, the area of the excavation and the cup/disc ratio) using spectral domain OCT (SD-OCT) [29]. They found that AL was significantly and strongly correlated with each examined parameter [29]. Mwanza et al. examined the effect of AL on the thickness of the ganglion cell layer and inner plexiform layer complex in the macula, also using SD-OCT [25]. Their results indicated that the thickness of the GCL+IPL complex decreased significantly by the increase in AL [25]. On the contrary, Ooto et al. did not find the same trend as the above authors for the correlation between AL and the thickness of any of the intraretinal layers using automatic segmentation and intraretinal layer thickness measurement on spectral domain OCT images [14]. It is worth to note that mild and high myopic eyes were excluded from the study by Ooto et al., while these were included in the study by Cheung et al. and Mwanza et al. [14, 25, 29]. Therefore, the explanation for the different results could at least in part be that the standard deviation of the AL of the examined eyes was very low in the study of Ooto et al., hence the significant deviations which are observable in the case of shorter or longer eyes did not affect their results.

Our results showed that the weighted mean thickness of the nuclear layers (GCL+IPL, INL and ONL) correlated with AL after adjustment for age, sex and image quality, the correlation getting stronger towards the outer layers. Compared to the above mentioned studies, in the present study the AL of the eyes was relatively in a wide range which could also contribute to our results.

According to two previous studies using OCT, the total thickness of the central, 1 mm diameter wide area of the macula (the central subfield) and total macular volume also correlate with AL, although with relatively low coefficients of correlation (r = -0.222 and r = 0.308, respectively) [30, 31]. Our results were in line with these findings as the correlation between total retinal thickness (a derivative of total macular volume) and AL was somewhat higher (r = -0.378) compared to the above mentioned studies.

It remains unknown whether thinning in axial myopia occurs equally in all retinal layers. Abott et al studied the changes in retinal thickness (in total and across layers) in a mammalian animal model (tree shrews, Tupaia belangeri) of high axial myopia using OCT and histological sections from the same retinal tissue. Analysis of retinal layers revealed that the inner plexiform, inner nuclear and outer plexiform layers are showing the most thinning. [32] From the biomechanic point of view, thinning of intermediate thinner layers in myopic eyes could be explained by stiffness conditions of the tissue exposed to mechanical stress with traction and shear forces acting at its innermost surface. [33]

Areal cell density measurements (cells/mm2) showed all neuronal cell types (photoreceptors, bipolar/horizontal cells, amacrine cells and ganglion cells) were involved in retinal thinning. [32] Our results are in accordance with the above; however, we also observed changes of the outer nuclear layer suggesting the additional involvement of the photoreceptors, as well. These changes in the outer retina may be mediated by fluid forces (e.g. active flows), such as the RPE active pump flux that creates a pressure-driven fluid flow between the choroidal space and the subretinal space. [34]

Wolsley et al. showed retinal thinning measured by OCT in human myopes compared to emmetropes along a line from 16° superior temporal to the fovea to 16° inferior nasal. The thinning appeared to slowly increase from 4° to 16° nasally and temporally, but regional differences were not analyzed in detail. Their possible explanation is the retinal laminar thickness change due to the shearing between retinal cell layers and cone packing. [35] The fact that the retinal thinning was more pronounced in the peripheral retinal layers correlates with our results that the correlations between AL and thickness of the retinal layers are stronger in the outer regions, perhaps due to the lower shear resistance of the thinner peripheral retina. [36–37]

The introduction of the latest SD-OCT devices led not only to a dramatic increase in mapping speed and also some increase in axial resolution, but the examination of the choroid became also possible. In the past years, promising results have been obtained by the manual segmentation and measurement of choroidal thickness on OCT images. Li et al. and Sogawa et al. demonstrated strong negative correlation between AL and choroidal thickness measured in the subfoveal area of young and healthy eyes (r = -0.624 and r = -0.735, respectively) [38, 39]. Unfortunately, it is not possible to obtain choroidal thickness from TD-OCT images due to the poor penetration and thus low resolution beneath the RPE, which is one of the shortcomings of our study.

As the growth of the eyeball is stipulated to continue until the age of 20 years [40], it is important to note that a longitudinal study spanning from adolescence to early adulthood would be necessary to evaluate the effect of axial length on the thickness of intraretinal layers of the macula under and above the age of 20 years. We hypothesize that as the eyeball stops to grow the nuclear layers follow the shape of an elongated globe and get thinner by lateral stretching, while the other layers are not capable of this stretching. It should, however, be taken into consideration with such a study that longer AL decreases the magnification of fundus imaging, making transverse dimensions appear smaller on the OCT scan, in inverse proportion to AL [41]. Unfortunately, in our study the number of subjects would have been low in both subgroups for such a focused analysis (n = 18 and n = 35 in the groups below and above 20 years of age, respectively) yielding statistical powers of 0.325 and 0.639 at a 5% significance level and therefore, we did not perform such subgroup tests to avoid type II error.

In conclusion, our study reveals the inverse correlation between the AL of the eyeball and the thickness of the intraretinal layers. However, there are some potential shortcomings of the study. Due to the limited retinal area of OCT imaging we could not assess whether the total volume of intraretinal layers remains constant despite variability in axial length. This could be achieved by enhanced and extended peripheral scanning of retinal tissue around the macula, which is not yet available technically. Another limitation is the lower resolution of the TD-OCT system and the number of interpolations used due to the six radial scans covering the macular area. First, we examined healthy eyes where no pathological retinal changes were anticipated that could negatively influence the precision of thickness measurements. Second, we have previously shown the high reliability of OCTRIMA measurements along with their comparability to SD-OCT thickness measurements [42–44]. Therefore, we believe that the same results would have been obtained by the implementation of an SD-OCT system, which was not available at the time of the study.

Based on our present results we suggest that the effect of AL should be taken into consideration when using OCT image segmentation techniques in future clinical studies involving adults while further studies are warranted to verify our observations in young subjects.

## Supporting Information

S1 Table Demographical data (age; gender; involved eye: right OD, left: OS) and manifest spherical equivalent refraction (MRSE), axial length (AL) and circumpapillary retinal nerve fiber layer (cpRNFL) of our study subjects. (XLS)

S2 Table Results of thickness (μm) measurements of intraretinal layers of our study subjects. The results organized by study regions. (Abbreviations: RNFL: retinal nerve fiber layer, GCL+IPL: ganglion cell and inner plexiform layer complex, INL: inner nuclear layer, OPL: outer plexiform layer, ONL: outer plexiform layer, RPE: retinal pigment epithelium, cpRNFL: circumpapillary retinal nerve fiber layer).
(XLS)
